# CBDB: The codon bias database

**DOI:** 10.1186/1471-2105-13-62

**Published:** 2012-04-26

**Authors:** Adam Hilterbrand, Joseph Saelens, Catherine Putonti

**Affiliations:** 1 Department of Biology, Loyola University Chicago, 1032 W Sheridan Road, Chicago, IL 60660, USA; 2 Bioinformatics Program, Loyola University Chicago, 1032 W Sheridan Road, Chicago, IL 60660, USA; 3 Department of Computer Science, Loyola University Chicago, 820 N Michigan Avenue, Chicago, IL 60611, USA

## Abstract

**Background:**

In many genomes, a clear preference in the usage of particular codons exists. The mechanisms that induce codon biases remain an open question; studies have attributed codon usage to translational selection, mutational bias and drift. Furthermore, correlations between codon usage within host genomes and their viral pathogens have been observed for a myriad of host-virus systems. As such, numerous studies have investigated codon usage and codon bias in an effort to better understand how species evolve. Numerous metrics have been developed to identify biases in codon usage. In addition, a few data repositories of codon bias data are available, differing in the metrics reported as well as the number and taxonomy of strains examined.

**Description:**

We have created a new web resource called the Codon Bias Database (CBDB) which provides information regarding the codon bias within the set of highly expressed genes for 300+ bacterial genomes. CBDB was developed to provide a resource for researchers investigating codon bias in bacteria, facilitating comparisons between strains and species. Furthermore, the site was created to serve those studying adaptation in phage; the genera selected for this first release of CBDB all have sequenced, annotated bacteriophages. The annotations and sequences for the highly expressed gene set are available for each strain in addition to the strain’s codon bias measurements.

**Conclusions:**

Comparing species and strains provides a comprehensive look at how codon usage has been shaped over evolutionary time and can elucidate the putative mechanisms behind it. The Codon Bias Database provides a centralized repository of look-up tables and codon usage bias measures for a wide variety of genera, species and strains. Through our analysis of the variation in codon usage within the strains presently available, we find that most members of a genus have a codon composition most similar to other members of its genus, although not necessarily other members of its species.

## Background

### Why does codon bias exist?

The redundancy within the genetic code accommodates a variable number of codons to encode for the same amino acid. Codon usage biases have been found to exist, ranging from relatively neutral to extremely strong [1-6]. Debate within the scientific community continues as to exactly why codon biases exist. Theories based on translational selection, mutational biases, and drift have all been found to contribute to codon biases [7-11]. Sequence-based analysis revealed that within organisms having a biased genome, the most frequently occurring codons often reflect the most abundant transfer RNA (tRNA) available [12-15]. Furthermore, through direct molecular manipulation protein-throughput was increased by designing coding sequences to utilize the most abundant tRNAs [16-18]. These bioinformatic and experimental studies suggest that translational selection may be the primary factor shaping codon usage.

Correspondences between codon usage and tRNA availability may signify selection for translational accuracy, selection for translational efficiency, or both [19-30]. Theories for selection for translational accuracy assume that the codon with the highest tRNA abundance has a lower missense error rate than its synonymous codons, considering tRNA gene copy numbers of both cognate and near-cognate tRNA abundances (e.g. [11,31]). If codon bias exists as a result of selection to maximize protein-throughput, codon usage often reflects available tRNAs. Because codon bias is often strongest within highly expressed genes and there exists a correspondence between generation time and the strength of codon bias within these highly expressed genes [6], it is believed that selection for translational efficiency plays some role in determining a species’ codon usage. Moreover, recent studies have shown that the usage of particular synonymous codons can also impact protein folding or misfolding [32-34].

As the abundances of individual tRNAs vary from one species to the next, so too do the preferences in codons. Translational selection seems most prevalent in viral species. Correspondence in virus-host codon usage has been observed in DNA-based, RNA-based, and retro-transcribing viruses (e.g. [35-39]). This observation is not isolated to eukaryote-infecting viruses; bacteriophages also exhibit codon biases similar to their host species (e.g. [40-42]). Given that viruses are heavily dependent upon their host for biosynthesis, utilizing the most prevalent tRNAs within the host would likely give the virus a translational advantage.

### Examining codon bias

In order to quantify the bias present within a species, a number of metrics have been developed. Calculating the codon usage frequencies within the genome can reveal biases, in particular when comparing these usage profiles to expected usage patterns and/or the usage profiles of synonymous codons, e.g. the frequency of preferred codons (FOP) metric [13], the synonymous codon usage order (SCUO) metric [43], and the %MinMax algorithm [44]. In order to compare the strength of biases within and between species, the relative synonymous codon usage (RSCU) and the geometric mean of the RSCU values, the codon adaptation index (CAI), were developed in which codon usage frequencies within highly expressed genes (HEGs) are specifically examined [6,23,45]. Numerous extensions of the CAI metric have been proposed, e.g. the self-consistent codon index (SCCI) [46] and relative codon adaption index (rCAI) [47]. Rather than looking at the codons themselves to ascertain biases, a second approach exists in which biases are assessed relative to individual tRNA abundances. The tRNA adaptation index (tAI), also inspired by the CAI metric, considers not only the gene copy number of the tRNA with the perfectly matched anticodon but also those tRNAs which can bind imperfectly [24]. The “local tAI” metric takes a similar approach, however the tAI measure is averaged for sliding windows across the gene rather than for the whole gene sequence [26]. In many species the codon usage in HEGs matches tRNA abundance [12,48].

Given the information encoded within the usage of codons, a number of tools and databases have been developed for analyzing the codon and tRNA content of genic and genomic sequences. CAIcal [49], CodonExplorer [50], and CodonW [51] calculate CAI values for user input sequences while E-CAI [52] calculates the expected CAI values by generating random sequences with a G+C content and amino acid composition similar to the user input sequence. The application CodonO [53] can analyze individual genomes or compare genomes using the SCUO metric. JCat [54] and GCUA [55] both calculate the RSCU values of user input sequences relative to a reference organism’s usage profile. The RSCU values for many species are contained in the Codon Usage Database [56]; this collection, however, does not appear to have been updated since June 15, 2007. The Microbial Genome Codon Usage Database [57] and Prokaryotes Codon Usage Database [58] have lookup tables of codon counts and frequencies for over 500 and 800 species, respectively (as of June 2011). The most comprehensive database of codon statistics for individual microbial strains can be found in the Codon Usage Bias Database (CUB-DB) [59]; individual links guide the user to individual strain values for many of the aforementioned metrics as well as two metrics developed by the database’s author [60].

### The codon bias database

We have developed a new web resource called the Codon Bias Database (CBDB) which lists RSCU, normalized RSCU, and frequency biases (FB) values for 300+ and counting bacterial strains. The genera selected for this first release of CBDB all have sequenced, annotated bacteriophages, thus providing a reference for those studying adaptation in phage. Following the metric proposed by Paul Sharp and collaborators [6,23,45], analysis is performed for the set of highly expressed genes (HEGs) and these gene sets have been manually curated. CBDB is organized in such a way to easily accommodate codon usage comparisons between strains and species.

## Construction and content

In this initial release, over 300 strains belonging to 17 genera were selected. The FASTA format (*.fna) and protein coding gene (*.ptt) files were downloaded from the NCBI FTP site [60]. Code developed in C++ generated *.ptt files containing only the annotations of the HEGs which includes 40 genes previously used for analysis of codon bias [6]; these genes encode for the translation elongation factor Tu (*tufA*), Ts (*tsf*) and G (*fusA*) as well as 37 ribosomal proteins (*rplArplFrplIrplT*, and *rpsBrpsT*). Each HEG file was then manually inspected to account for variations in naming conventions. The HEG *.ptt files, which include information regarding the location of the gene within the genome, the gene name, and information about the protein product, are available for download for each strain.

Using code developed in C++, the frequency of each codon within each strain’s set of HEG sequences was calculated. These frequencies were then used to calculate three metrics representing the codon usage patterns: the relative synonymous codon usage (RSCU), normalized relative synonymous codon usage (NRSCU) and frequency bias (HEG FB) values. The RSCU metric, introduced by Sharp et al. [45], is the observed frequency of the codon divided by the frequency expected under the assumption of equal usage of synonymous codons. The commonly used metric of quantifying codon bias, the CAI value, is derived by referencing the set of RSCU values for a species’ HEG sequence set. RSCU values can vary from zero to the number of synonymous codons available for a particular amino acid. The NRSCU value scales the RSCU values such that each codon’s value is between zero and one. Thus, each amino acid is weighted equally while still retaining the variance in usage between synonymous codons. The last metric, the HEG FB metric, is the relative frequency of the codon within the set of all codons present in the HEG sequences. The FB values also reflect the variation in synonymous codon usage, but in contrast to the NRSCU metric it also captures biases in amino acid usage within the HEG set. These metrics are calculated as follows:

(1) $$RSCU_{\mathrm{ij}}=\frac{x_{\mathrm{ij}}}{\frac{1}{n_{i}}\sum_{j=1}^{n_{i}}x_{\mathrm{ij}}}=\;NRSCU_{\mathrm{ij}}=\frac{x_{\mathrm{ij}}}{\sum_{j=1}^{n_{i}}x_{\mathrm{ij}}}=\;HEG\;FB_{\mathrm{ij}}=\frac{x_{\mathrm{ij}}}{\sum_{i=1}^{20}\sum_{j=1}^{n_{i}}x_{\mathrm{ij}}}$$

where *x*_*ij*_ is the number of occurrences of the *j*^th^ codon for the *i*^th^ amino acid and *n*_*i*_ is the number of synonymous codons which encode for the *i*^th^ amino acid. The user can download a table containing the results of these calculations for each strain available through CBDB as well as a table containing these values for all strains of the selected genus.

The nucleotide and protein sequences of the HEGs for each strain are also available for download from CBDB in FASTA format.

## Utility & discussion

The motivation behind creating the CBDB site was two-fold. Firstly, this site was designed to provide a resource of codon frequency look-up tables for researchers who are investigating translational selection and codon bias in bacteria. Researchers can assess if a nonsynonymous mutation observed is for a codon which is more/less preferred. Secondly, a number of groups, including our own, utilize the phage-host system to explore viral evolution. In particular, CBDB is a resource for recognizing selection within evolving species.

While CUB-DB [59] includes much of the same data included in CBDB, we wanted to present the data in such a way that one could easily compare the variation in biases between strains and species within a genus. Secondly, CBDB also contains the FASTA format sequences of the highly expressed genes as well as the gene annotation information, which could then be subsequently analyzed by any of the aforementioned codon analysis software tools. Thus, researchers can go to one repository for this data. In contrast with the resource of the Highly Expressed Genes Database or HEG-DB [61], CBDB includes only the set of HEG identified by Sharp et al. [6] and the calculation of CAI values. Furthermore, this initial release of CBDB contains more species than is available through HEG-DB [61].

**Figure 1:**
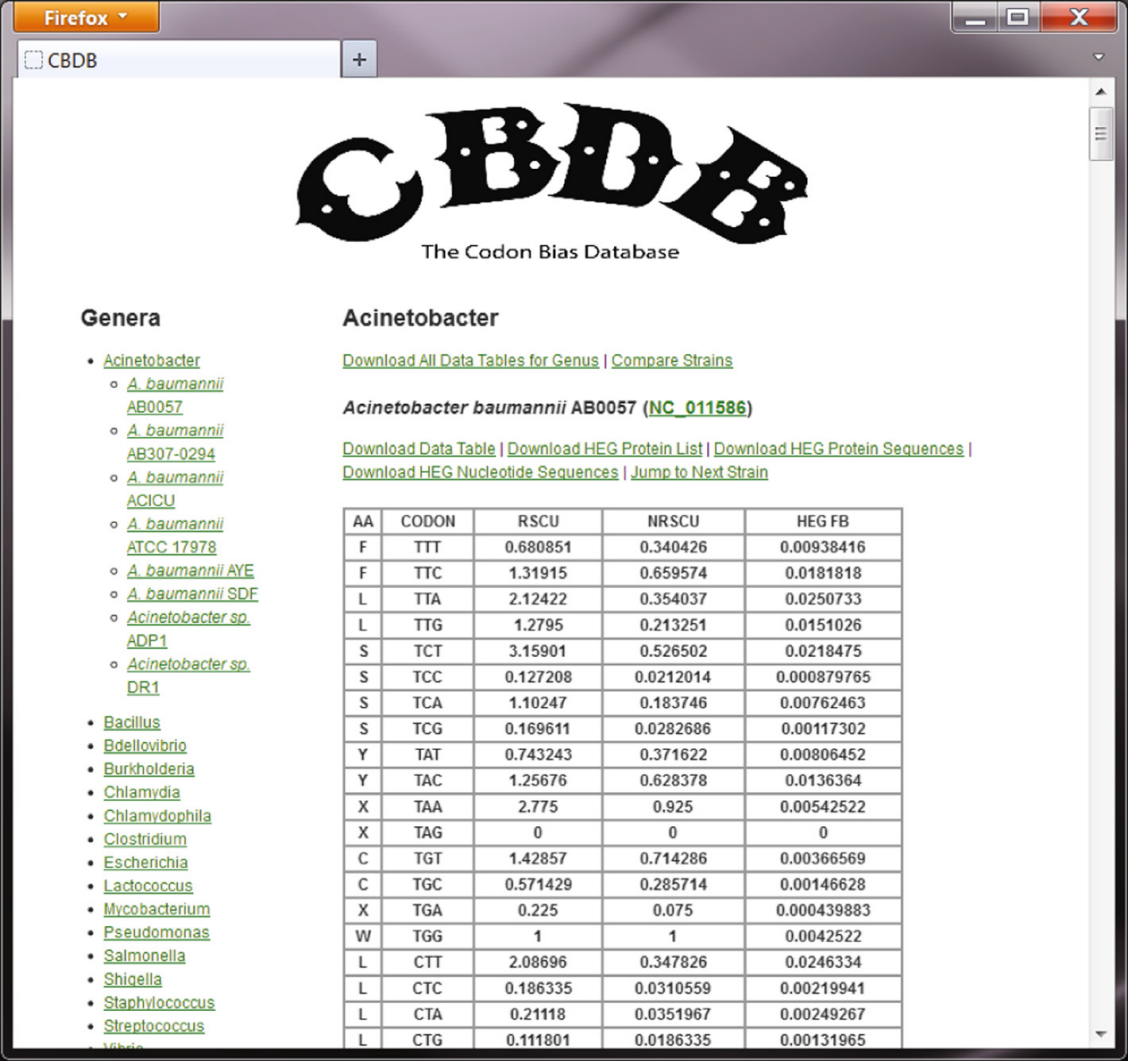
**CBDB interface, the results for the first species of the *Acinetobacter *genus.**

Figure1 shows a screen shot of the CBDB website, showing the results for the first strain listed for the genus *Acinetobacter*. Each strain’s name and NCBI RefSeq number, which includes a hyperlink to the NCBI genome record, is listed followed by links to download: the data table (format *.xls), the tab-delimited gene annotation file for the HEG (format *.ptt), the FASTA format amino acid sequences of the HEG set, and the FASTA format nucleotide sequences of the HEG set. Below these links is the data table containing the amino acid abbreviation for each codon and each codon’s RSCU, NRSCU, and HEG FB values. At the top of each genus’ page is a link to download all of the data tables in a single MS Excel document. Also included is a link to a single MS Excel file which contains the RSCU values for each codon for each strain and an interactive graph to compare biases between strains. The list of all of the available genera appears in the navigation on the left pane of each page and all of the species available for a selected genus. Moreover, the navigation pane includes a link describing the codon bias metrics used and associated references.

While in this release of the database only 300+ species are available, additional species will be added on a regular basis; all of the annotated bacterial species available through NCBI will be included over the coming months. Furthermore, we are in the process of developing and making publicly available through the site functionality for conducting inter- and intra-species as well as phage-host comparisons of codon usage profiles.

### Exploring codon bias with CBDB

**Figure 2:**
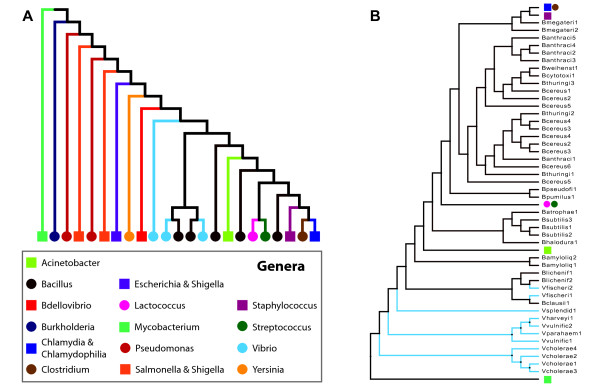
**Visualization of similarity/dissimilarity of codon usage biases between strains and species. (A) **The majority of species appears most similar to other species/strains within their genus and thus is represented by a single branch in the tree. **(B)** Visualization of the *Bacillus *strains and their placement in the tree; several different species form their own clades.

Previous analysis of the variation in codon usage between bacterial species selected just a few representatives of a genus and species [6]. We were interested to see how codon usage varied between species in the same genus as well as between different strains of the same species. For each of the three metrics included in CBDB, we calculated the correlation in codon usage for each pair of strains. A distance matrix was then computed as (1-r)/2 such that the instance of a pair being anticorrelated has a distance of 1 and a perfectly correlated pair has a distance of 0. The FITCH application of the PHYLIP package [62] was used to derive a tree to visualize similarities/dissimilarities in codon usage. Figure2A shows the tree derived when the NRSCU values were compared for all strains. (Clades were collapsed when the subtree contained only one genus. *Shigella* was found to group both with *Escherichia* and *Salmonella*. *Chlamydia* and *Chlamydophila* were also collapsed into single nodes as some strains were closer to strains of the other species than of their own species.) From Figure2A, we also identified two branches including *Pseudomonas* (maroon circle) and *Salmonella & Shigella* (orange square) species. The leftmost *Salmonella* &*Shigella* branch, separating the pseudomonads is a single species – *Salmonella enterica* subsp. arizonae serovar 62:z4,z23:-- str. RSK2980 (GenBank: NC_010067). Also from this visualization of codon usage bias we noticed that the 34 *Bacillus* strains are interspersed amongst other species in the tree. The two *B. lincheniformis* and single *B. clausii* strain show a more similar codon usage pattern to the *Vibrio fisheri* sequences than they do to the other bacilli, as is shown in Figure2B. Looking at the *Bacillus* genus data file available from CBDB which includes statistics for all of the strains, one can see variation in usage between species.

**Figure 3:**
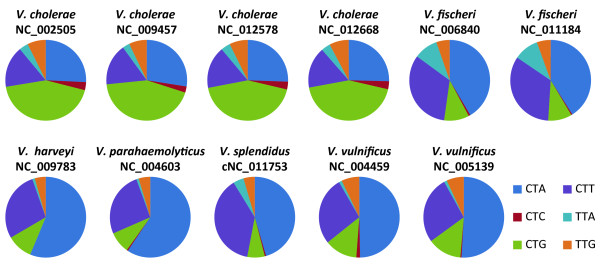
**Variation in the usage of Leucine synonymous codons in *Vibrio *species.**

The genera included in this first release vary in the strength of their bias. For instance, as has been previously documented [6], the *Chlamydiae* phylum does not exhibit a significant bias. The eight *Chlamydia* and *Chlamydophila* species examined here do not show a strong bias. ANOVAs were performed for all three statistics revealing that the variation between the species is not statistically significant. The *Vibrio* species, however, are strongly biased [6] and exhibit differences in codon usage between species. For instance, Figure3 shows the biases for the 11 *Vibrio* species examined for the six leucine codons. Referring to the Genomic tRNA database [63], one can find that the non-*cholerae* species have more TAG-anticodon tRNAs (blue) than any other Leucine tRNAs. This thus can explain the preference within these species for CTA codon. The *V. cholerae* species also have more TAG-anticodon tRNAs than any other Leucine tRNAs, but in contrast to the non-*cholerae* species they also have CAG-anticodon tRNAs (green) [63].

## Conclusions

As recent research has found, the use of particular codons can improve translational accuracy and efficiency in addition to serving as a signal for co-translational protein folding [11,19-34]. Comparing usage between species and strains can expose variation in usage between closely related organisms. Furthermore, this site serves as a resource for studying the mechanisms shaping codon usage within bacteriophages. The Codon Bias Database (CBDB) provides a centralized repository of look-up tables and codon usage bias measures for a wide variety of genera, species and strains, facilitating comparisons in codon usage between closely related species such as the one presented here.

## Availability and requirements

The Codon Bias Database (CBDB) is freely available without restriction at http://www.cbdb.info. This website has been tested with browsers of Safari, Internet Explorer and Firefox.

## Competing interests

The authors declare that they have no competing interests.

## Authors contributions

AH and JS collected the data as well as participated in the design of the site and writing of the manuscript. CP conceived of the study and site and developed the code used for generating data and analyses in addition to participating in the design of the site and writing of the manuscript. All authors read and approved the final manuscript.
